# Assessment of central nervous system vasculitis in children based on high-resolution vascular wall imaging

**DOI:** 10.1093/rap/rkae038

**Published:** 2024-03-07

**Authors:** Yimin Cao, Yue Sun, Zexi Yi, Weixin Meng, Xueying Zhao, Xuran Feng, Pingyong Feng, Sicong Wang, Mingfeng Zhang, Lixia Zhou

**Affiliations:** Department of Medical Imaging, The Second Hospital of Hebei Medical University, Shijiazhuang, China; Department of Medical Imaging, The Second Hospital of Hebei Medical University, Shijiazhuang, China; Department of Medical Imaging, The Second Hospital of Hebei Medical University, Shijiazhuang, China; Department of Medical Imaging, The Second Hospital of Hebei Medical University, Shijiazhuang, China; Department of Medical Imaging, The Second Hospital of Hebei Medical University, Shijiazhuang, China; Department of Medical Imaging, The Second Hospital of Hebei Medical University, Shijiazhuang, China; Department of Medical Imaging, The Second Hospital of Hebei Medical University, Shijiazhuang, China; GE Healthcare, MR Research China, Beijing, China; Rheumatology and Immunology Department, The Second Hospital of Hebei Medical University, Shijiazhuang, China; Department of Medical Imaging, The Second Hospital of Hebei Medical University, Shijiazhuang, China

**Keywords:** central nervous system vasculitis, children, HR-VWI, MRI

## Abstract

**Objectives:**

Central nervous system vasculitis (CNSV) is a rare disease. High-resolution vessel wall imaging (HR-VWI) enables the identification of inflammatory changes within the vessel wall. Few studies have applied HR-VWI to assess CNSV in children. This study delves into the utility of HR-VWI for diagnosing and treating CNSV in children, with the aim of enhancing clinical diagnosis and efficacy evaluation.

**Methods:**

Imaging data were acquired from children who underwent HR-VWI examinations. The study meticulously analysed clinical data and laboratory tests to discern the characteristics and distribution patterns of diverse vasculitis forms.

**Results:**

In children, CNSV mainly involves medium vessels with grade 1 and 2 stenosis (grade 4 stenosis is rare), and the imaging features generally show centripetal and moderate enhancement, suggesting that this feature is specific for the diagnosis of CNSV. High-grade stenosis, concentric enhancement and strong enhancement of the vasculature indicate more severe disease activity. Remarkably, HR-VWI proved to be significantly more sensitive than magnetic resonance angiography in detecting CNSV. Among the 13 cases subjected to imaging review, 8 demonstrated a reduction or resolution of vessel wall inflammation. In contrast, five patients exhibited worsening inflammation in the vessel wall. HR-VWI demonstrated that changes in vessel wall inflammation were closely correlated with changes in brain parenchymal lesions and symptoms.

**Conclusion:**

This study underscores the diagnostic value of HR-VWI in CNSV assessment and treatment monitoring, offering a quantitative evaluation of CNSV in children.

Key messages

- This study summarizes the high-resolution vascular wall imaging (HR-VWI) characteristics of central nervous system vasculitis (CNSV) in children.
- HR-VWI can evaluate the activity of CNSV in children.
- The diagnostic power of HR-VWI was higher than that of magnetic resonance angiography for CNSV in children.

## Introduction

Central nervous system vasculitis (CNSV) can be divided into primary angiitis of the central nervous system (PACNS) and secondary central nervous system vasculitis (SCNSV) [1]. SCNSV is an inflammatory vascular lesion involving the brain and spinal cord, with a complex and varied clinical presentation, mostly showing focal or diffuse neurological impairment and lacking specific diagnostic indicators [2, 3]. The commonly used imaging methods to show cerebral vasculitis include digital subtraction angiography (DSA), magnetic resonance angiography (MRA) and computed tomography angiography (CTA). DSA is considered the gold standard for the diagnosis of CNSV, which is typically characterized by alternating stenosis and dilatation of blood vessels approximating bead-like changes. None of the above exams can well show inflammation in the wall of the vessel, MRA has poor sensitivity to CNSV and there is radiation damage in DSA and CTA [4, 5] and thus should not be used for CNSV in children.

High-resolution magnetic resonance vessel wall imaging (HR-VWI) is a non-invasive technique that allows direct observation of the arterial lumen and vessel wall and is used in the study of atherosclerotic plaques and related cerebral ischaemia [6]. Reports on HR-VWI for the diagnosis of CNSV in adults have gradually increased, but there are fewer imaging studies of HR-VWI for CNSV in children. The aim of this article is to investigate the value of HR-VWI in CNSV in children.

## Methods

### Baseline data

This was a retrospective study. Patients ≤18 years of age who underwent HR-VWI examination in our hospital from October 2019 to May 2023 were diagnosed with CNSV based on clinical symptoms and signs, neuroimaging and laboratory examinations. For the purpose of this study, CNSV was defined as an inflammatory vasculopathy of cerebral arteries, either restricted to the CNS or as part of a systemic disease. The latter included CNS manifestations of systemic autoimmune-mediated vasculitis. We evaluated the available imaging examinations (HR-VWI, MRI and MRA) for the presence of intracranial vascular inflammation and corresponding pathological vascular features [1, 3]. The study was authorized by the Ethics Committee of the Second Hospital of Hebei Medical University and was performed in line with the principles of the Declaration of Helsinki. All patients participating in the study provided written informed consent to use their clinical data for research purposes.

### MRI scheme

The HR-VWI sequences were examined using a SIGNA Architect 3.0 T MRI scanner (GE Healthcare, Chicago, IL, USA) and an Achieva 3.0 T MRI scanner (Philips Healthcare, Andover, MA, USA) and the HR-VWI sequences were CUBE: TR 850 ms, TE 15 ms, TE flip angle 90°, refocusing control angle 50°, layer thickness 2 mm, matrix 200 mm × 181 mm; VISTA: TR 800 ms, TE 20 ms, flip angle 90°, refocusing control angle 50°, layer thickness 2 mm, matrix 200 mm × 181 mm. Other imaging protocols included T1 weighted image, T2 weighted image, T2 FLAIR, diffusion-weighted imaging (DWI) and MRA.

### Image data evaluation

All images were reconstructed and processed at the post-processing workstation. Two experienced imaging physicians conducted independent double-blind analysis of the obtained MRIs. In case of disagreement, a consensus was reached through consultation.

All cases were counted according to the affected segments of the blood vessels [5]. The large and middle cerebral vessels were divided into 11 segments, including bilateral internal carotid arteries (ICAs), bilateral anterior cerebral arteries (ACAs), middle cerebral arteries (MCAs), posterior cerebral arteries (PCAs), bilateral vertebral arteries (VAs) and basilar arteries (BA). Suspected small vessel lesions were identified through CURVE （A MRI post-processing technique). We combined MRA and HR-VWI to estimate the degree of vascular stenosis and grade: 1 = mild stenosis of the lumen (estimated stenosis grade <30%); 2 = moderate stenosis (30–69%); 3 = severe stenosis (70–99%); 4 = blocked.

For each depicted steno-occlusive lesion, the degree of vessel wall contrast enhancement was documented as either 0 = no enhancement, 1 = moderate enhancement or 2 = strong enhancement, as defined and shown by Pfefferkorn *et al*. [7]. Enhancement was recorded as concentric if it was uniform and involved the entire circumference of the arterial wall and as eccentric if it was non-uniform, mainly on one side of the arterial wall and not involving the entire circumference. Disease activity was assessed in combination with expert consensus [8–10], or if no written criteria were available, a combination of clinical and imaging conditions were assessed. Follow-up MRI was classified into three levels based on changes in brain parenchymal lesions and vascular wall inflammation as remission, stability or progression.

## Results

A total of 36 patients, consisting of 18 males and 18 females, with a mean age of 15.0 years (s.d. 3.3), were recruited for this study. The study population comprised 6 cases of Takayasu arteritis (TA, all involved segments C5–7 of the internal carotid artery) and 23 cases of secondary CNSV, including 8 cases of SLE, 2 cases of SS, 4 cases of autoimmune demyelination, 5 cases of UCTD, 4 cases of viral infection and 7 cases of vasculitis of undetermined aetiology (VUA). A total of 26 patients were diagnosed with large/medium vessel CNSV, 6 patients had small vessel CNSV and 4 patients had both types of vasculitis (large/middle and small vessel), as shown in Tables 1 and 2.

**Table 1.** Patient details

Characteristics	Values
Age, years, mean (s.d.)	15.0 (3.3)
Male, *n* (%)	18 (50.0)
Female, *n* (%)	18 (50.0)
Symptom, *n* (%)	
Acute stroke, *n* (%)	14 (38.9)
Epilepsy, *n* (%)	2 (5.6)
Headache, dizziness, *n* (%)	7 (19.4)
Fever, butterfly rash, joint swelling, *n* (%)	6 (16.7)
Other (blurred vision, involuntary movements of hands, etc.), *n* (%)	7 (19.7)
Abnormal CSF, *n* (%)	27 (75.0)
MRI lesion distribution, *n* (%)	
Supratentorial–cortex and white matter	21 (58.3)
Supratentorial–deep grey matter nuclei	11 (30.6)
Subtentorial–brainstem	7 (19.7)
Infratentorial–cerebellar hemisphere	5 (13.9)
No obvious lesions	7 (19.4)
HR-VWI positive	36 (100.0)
MRA positive	16 (44.4)
MRA negative	20 (55.6)

**Table 2.** Various sizes of diseased vessels corresponding to different aetiologies

Vessel size	Age, years, mean (s.d.)	Sex	TA, *n*	SLE, *n*	SS, *n*	AD, *n*	UCTD, *n*	VI, *n*	VUA, *n*
Large	15.3 (3.9)	M: 3, F: 3	6	–	–	–	–	–	–
Medium	15.9 (2.5)	M: 5, F: 9	–	2	2	3	3	2	2
Small	12.0 (4.4)	M: 6	–	2	–	2	–	–	2
Multiple	15.4 (1.6)	M: 4, F: 6	–	4	–	1	–	2	3

AD: autoimmune demyelination; F: female; M: male; VI: viral Infection.

**Figure 1.**
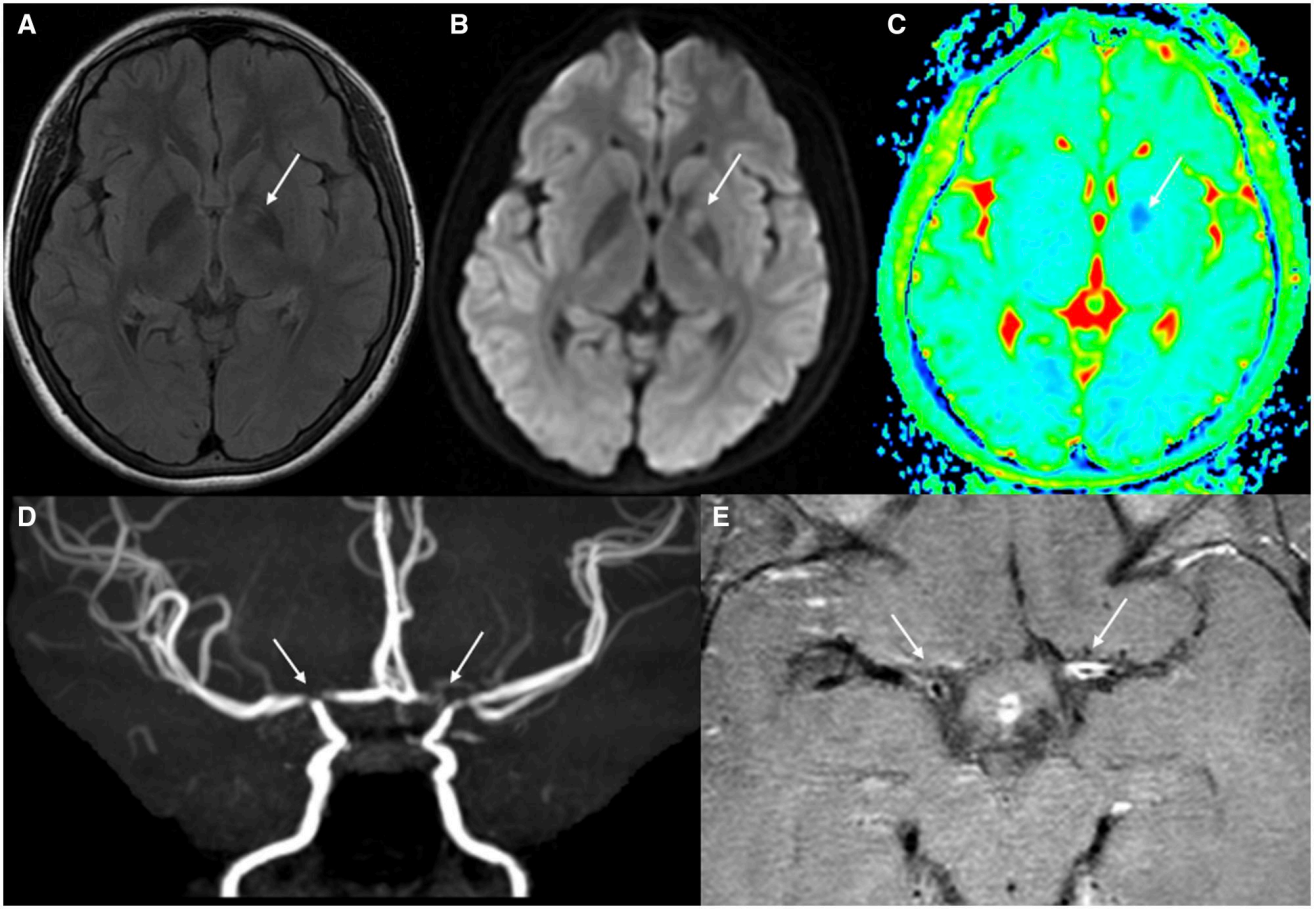
A 15-year-old female SLE patient. Right limb weakness with slurred speech for 15 days, ANA and aPL antibody positive. MRI: **(A)** T2-weighted FLAIR, **(B)** DWI, **(C)** ADC; acute infarction in the left basal ganglia. **(D)** MRA: severe stenosis of the lumen of the bilateral ICA C7 segments, bilateral ACA A1 segments and bilateral MCA M1 segments. **(E)** HR-VWI: bilateral ICA C7 segments, bilateral ACA A1 segments, bilateral MCA M1 segments with wall thickening, high enhancement and stenosis grade 3

MRI results showed that the parenchymal brain lesions in large/medium vasculitis (21/26 cases) showed foci of acute and chronic ischaemia, haemorrhage and secondary demyelination in the cerebral cortex, white matter, brainstem and cerebellar hemispheres in the area of the responsible vessel (Fig. 1). Cases of small vessel vasculitis (5/6 cases) presented with foci of ischaemia or demyelination in the basal ganglia region, thalamus and hippocampus. Multiple forms of parenchymal brain lesions were seen in the cases of large/medium and small vasculitis (3/4 cases).

**Figure 2.**
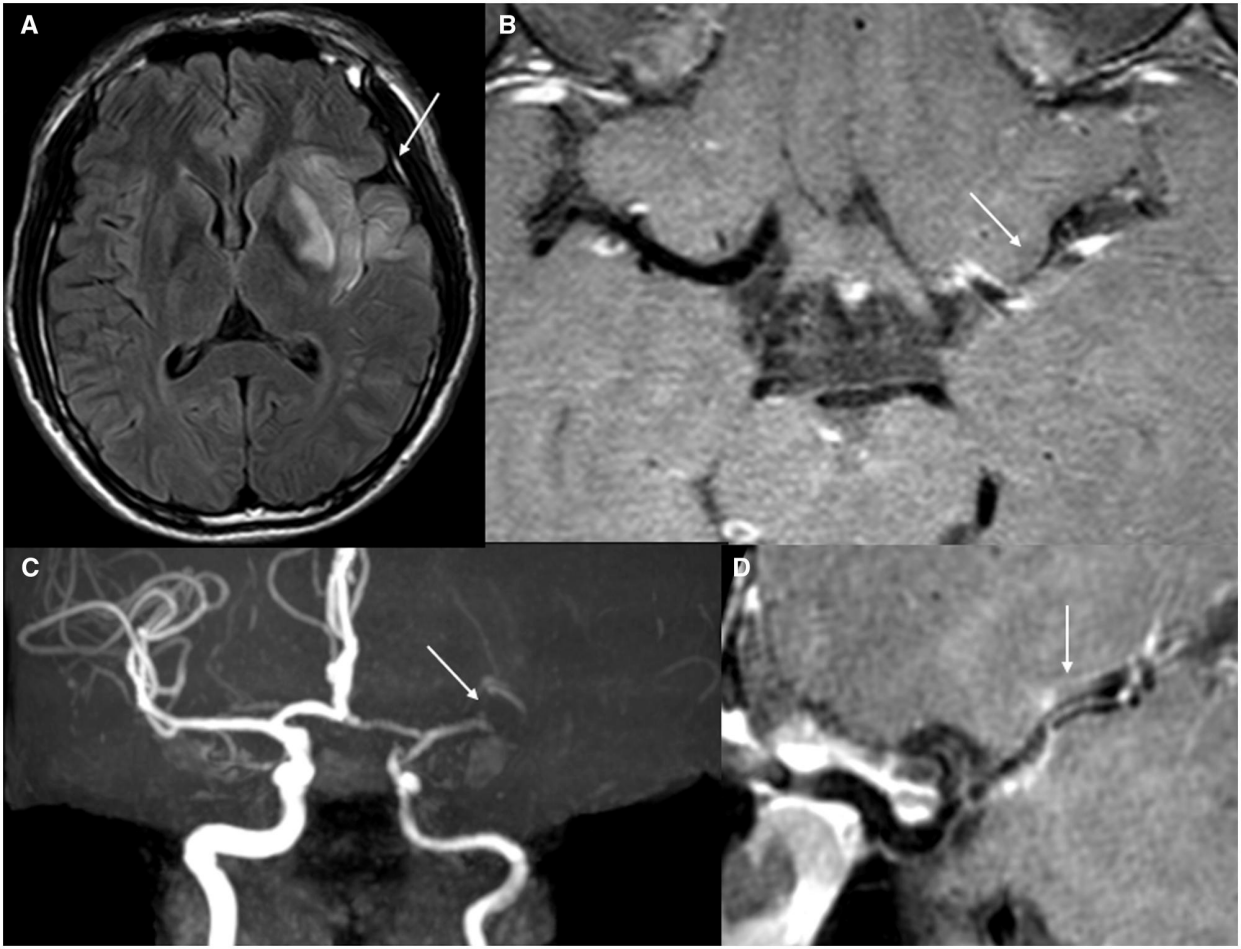
An 18-year-old male UCTD patient. Right limb weakness for 4 days admitted with ANA positive. **(A)** Left temporal lobe, insula and basal ganglia infarction with oedema. **(B)** Diffuse stenosis of the left ICA, with a prominent C7 segment and reduced distal branching of the left middle cerebral artery stenosis. **(C, D)** Left MCA M1 segment with wall thickening and highly eccentric enhancement, with grade 4 lumen stenosis

**Figure 3.**
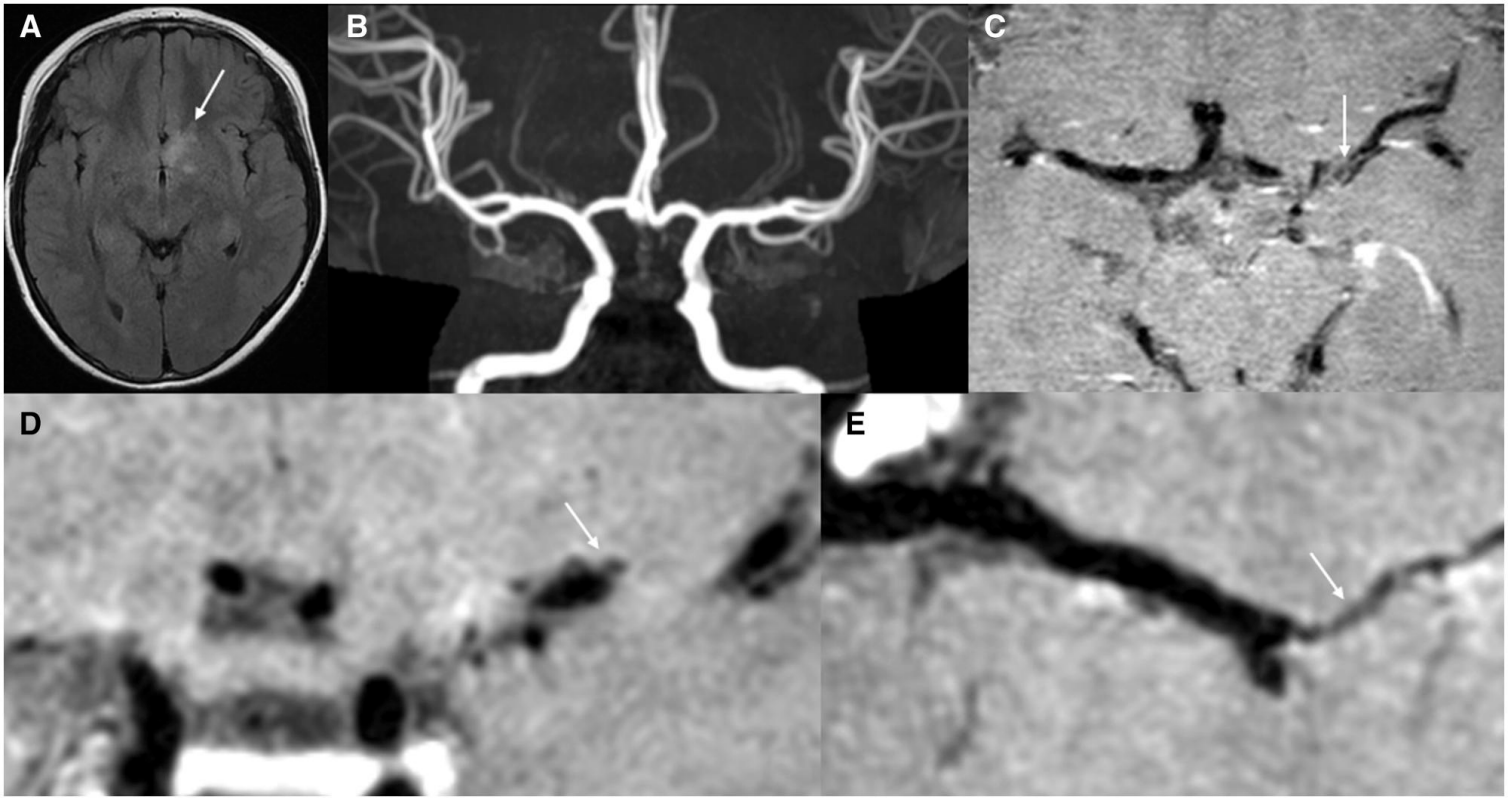
A 10-year-old male MOGAD patient. Involuntary movements of the hands for 3 days. He was admitted to the hospital with serum MOG IgG positive. **(A)** Paraventricular demyelinating lesion in the third ventricle. **(B)** MRA shows no abnormality. **(C–E)** Mild thickening of the wall of the perforating artery with marked enhancement in the left middle M1 segment of the brain

By HR-VWI, 89 of 396 large and medium vessels were implicated. Anterior circulatory vessels were involved in 9 patients, posterior circulatory vessels in 10 patients and both anterior and posterior circulations in 11 patients. A total of 26 cases had numerous segmental inflammatory foci and 4 cases had vasculitis in a single segment. The remaining 6 cases involved only small blood vessels (Figs 2 and 3).

There were 20 large vessel segments and 69 medium vessel segments among the involved vessel segments. A total of eight large vessel segments were involved in TA patients (grade 1 stenosis was present in four cases, grade 2 in two cases and grade 3 in two cases), all of which had internal carotid artery C5–7 segment lesions. After assessment, six TA patients were in the active phase of the disease. SCNSV had a total of 8 large vessel segments involved (grade 1 in 6 cases and grade 3 in 2 cases), 52 medium vessels involved (grade 1 in 34 cases, grade 2 in 11 cases, grade 3 in 26 cases and grade 4 in 1 case) and pure small vessel involvement was present in 4 patients. Two patients were moderately active and six patients were severely active for SLE and one patient was moderately active and one patient was severely active for SS. All other patients were in the active phase of the disease by combined clinical and imaging assessment. A total of 4 large vessel segments were involved in patients with UE (grade 2 in 1 case, grade 3 in 2 cases and grade 4 in 1 case), 17 medium vessels were involved (grade 1 in 4 cases, grade 2 in 5 cases, grade 3 in 7 cases and grade 4 in 1 case) and 2 patients had simple small vessel involvement. All seven VUA patients were in the active phase of the disease.

Vessel wall enhancement, in varying degrees, was present in all affected segments (100%), with 47 (52.8%) of the diseased vessel segments being moderately enhanced and 42 (47.2%) of the diseased vessel segments being highly enhanced. The majority of vessel wall enhancement was centripetal, with 81 cases exhibiting this pattern, while only 8 cases showed eccentric enhancement. All patients with concentric and strongly enhanced blood vessels had more severe disease activity than those with eccentric and moderately enhanced blood vessels. Among patients with small vessel vasculitis, seven small vessel lesions exhibited moderate enhancement and three showed significant enhancement. However, due to low resolution, accurately assessing the degree of stenosis was difficult.

The above results indicate that patients with high-grade stenosis and strongly enhanced concentric blood vessels have more severe disease activity than those with low-grade stenosis and moderately enhanced eccentric blood vessels.

A total of 20 patients were MRA negative, with no signs of diseased vessels. HR-VWI showed 36 diseased vessels with grade 1 in 31 cases and grade 2 in 5 cases, with an average degree of stenosis of grade 1.1, all with moderate enhancement. Small vessel CNSV was present in six patients and both large/medium vessel and small vessel CNSV were present in two patients. A total of 25 of the above lesions were moderately enhanced and 11 were highly enhanced.

**Figure 4.**
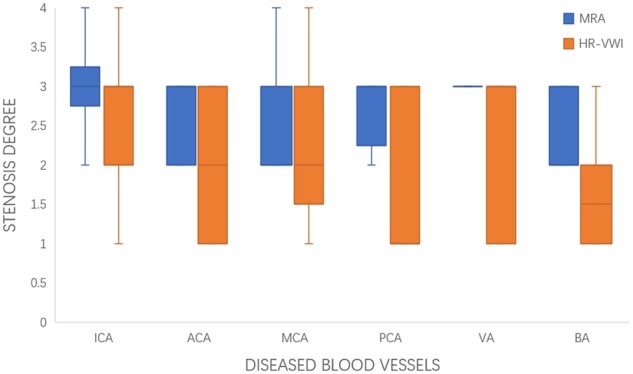
MRA positive *vs* HR-VWI

A total of 16 cases were MRA positive. MRA showed 32 diseased vessel segments with grade 1 in no cases, grade 2 in 10 cases, grade 3 in 19 cases and grade 4 in 3 cases, with an average degree of stenosis of 2.8. After re-evaluation by HR-VWI, 21 additional diseased vessels were identified, for an overall detection of 53 diseased vessels with stenosis of grade 1 in 17 cases, grade 2 in 14 cases, grade 3 in 19 cases and grade 4 in 3 cases, with an average degree of stenosis of 2.2. A total of 22 of the above lesions were moderately enhanced and 31 were highly enhanced (Fig. 4).

In the comparison between groups, the degree of stenosis and enhancement was significantly higher in MRA-positive patients than in MRA-negative patients (*P* < 0.05). HR-VWI exhibits considerably higher detection rates and accuracy in children with CSNV compared with MRA (*P* < 0.05).

Steroid and/or immunosuppressive therapy, along with symptomatic treatment, were administered to 36 patients during their hospitalization, resulting in varying degrees of remission. The medical records of 13 patients were reviewed over a period of 24 months, comprising 11 cases of large/medium vessel vasculitis and 2 cases of small vessel vasculitis. In 8 of 13 cases, a decrease in the extent of inflammation in the vessel wall and a reduction in wall thickening and enhancement was demonstrated. The average decrease in luminal stenosis was grade 0.5, while the degree of wall enhancement decreased by 62.4%, and clinical symptoms were alleviated (Supplementary Fig. S1, available at *Rheumatology Advances in Practice* online). Additionally, two patients with small vessel vasculitis did not exhibit distinct diseased vessels. Eight patients had re-examined cerebrospinal fluid (CSF) tests that were negative. After clinical evaluation, these patients were found to be in a non-active phase and had a good recovery. In 5 of 13 cases, the disease progressed, resulting in an increase in inflammation within the vessel wall as well as worsening of wall thickening and enhancement by HR-VWI (Supplementary Fig. S2, available at *Rheumatology Advances in Practice* online). Furthermore, three new diseased vessel segments were detected, and the overall luminal stenosis increased by an average grade of 0.9 and wall enhancement by 40.0%. The disease was in an active phase. The inflammatory markers of CSF continued to increase in the above patients after re-examination. After discharge, the remaining 23 patients did not agree to undergo imaging follow-up. They reported remission during clinical follow-up and no recurrence during the follow-up period.

## Discussion

HR-VWI has high spatial resolution and soft-tissue contrast, and application of the black-blood method to suppress the blood signal in the vessel lumen yields a clear image of the vessel wall. Scanning after contrast injection can be used to assess the enhancement characteristics of vessel wall lesions and determine the degree of luminal stenosis [11, 12]. MRA has a low sensitivity (36.0%) and only shows stenosis of more than grade 2.8 and is easily missed for stenosis of less than grade 1.1. CNSV lesions have increased endothelial permeability due to inflammation, which can be detected by contrast. Due to the increase in endothelial permeability in CNSV, the contrast agent leaks from the lumen into the arterial wall, which indicates the disease activity. HR-VWI has become an important imaging tool to monitor the degree of vasculitis activity in adults with CNSV and to judge the efficacy of the treatment [9]. However, there are fewer reports of studies related to HR-VWI of CNSV in children and there is a lack of observations and follow-up of a large number of cases. This study reported 36 cases of CNSV in children [13–16].

In this study, we found that HR-VWI of CNSV in children has certain imaging characteristics. Inflammation of the vessel wall in children with CNSV shows predominantly centripetal enhancement, with a small proportion showing eccentric enhancement. Enhancement is moderate to high during active inflammation and decreases during remission. This is similar to previous reports in adult vasculitis [17]. Most CNSV in this group of children had mild–moderate stenosis, with a smaller proportion of severe stenosis and occlusion of the lumen (4/36 cases) [18].

After hormonal and immunosuppressive therapy, clinical and imaging remission was faster and review MRI or HR-VWI suggested that the brain parenchyma and vasculitis were also in varying degrees of remission, suggesting that children’s vasculitis had a better response to recent treatment. The progression of brain parenchymal lesions and vasculitis during follow-up in some patients was thought to be related to a lack of regular treatment or inadequate doses of medication taken after discharge from the hospital.

CNSV in children has been reported in the literature to be predominantly large/medium vessel lesions, with some small vessel involvement. There is no significant difference between the anterior and posterior circulation in those with more multisegmental vascular lesions than those with single segments. The vascular involvement in our group of children with CNSV is similar to that reported in the literature [19]. In contrast to childhood CNSV, adult CNSV has significantly more lesions in the anterior circulation than in the posterior circulation [20]. The vascular condition evaluated by HR-VWI is consistent with the activity of CNSV. HR-VWI can be used to assess disease activity when the clinical picture is unclear and to inform diagnosis and treatment. In our enrolled cases, TA patients had abnormalities in different rheumatoid antibodies and exhibited significant lesions in the C5–7 segment of the internal carotid artery. We speculate that this may also be one of the phenotypes of SCNSV.

Parenchymal lesions in children with CNSV present as acute strokes in areas supplied by inflammatory vessels, especially in those with large/medium vessel involvement. Small vessel involvement often manifests as small chronic ischaemic foci, demyelination-like foci or inflammation-like foci in the brain parenchyma, often in the basal ganglia, thalamus, hippocampus, pons and other areas of penetrating vascular supply. All patients with small vessel vasculitis in our paediatric patients were male, whereas the prevalence of large and medium vessel vasculitis was higher in females than in males, which may be related to the bias of the results due to the small number of cases and needs to be confirmed by a large number of cases [3, 21].

Cerebral small vessel vasculitis is challenging to detect and diagnose in children, with a significant rate of underdiagnosis. Accurate imaging localization and image post-processing may increase the rate of positive diagnoses, particularly with MR equipment with a higher field strength. Eiden et al. [6] supports this point. In this study, there were 10 cases of cerebral small vessel lesions where the penetrating arteries of the anterior circulation were found to be involved more often than those of the posterior circulation. As a result, brain parenchymal foci occurring in the basal ganglia were more frequent. These findings are consistent with previous research.

The majority of cases within our cohort were linked to immune disorders, with some variation in vessel involvement depending on the specific disease [18]. Our research reveals that TA chiefly affects large vessels, such as the internal carotid and basilar arteries, with infrequent inflammation in smaller vessels. SLE, SS and UCTD are conditions that can involve vessels of varying sizes. In the overall SCNSV population, moderate vascular involvement was most common, which is in line with the existing literature [22, 23]. In addition, CNSV is closely linked to viral infections and can be a result of the varicella zoster virus, herpes simplex virus (HSV) or cytomegalovirus [24, 25]. In this study, HSV infection was prevalent (serum HSV Ig positive), and CNSV resulting from viral infections mainly affected the medium vessels of the brain (in three of four cases), while the involvement of large and small vessels was less frequent. In this study, seven patients presented with CNSV of unidentified origin. All clinical examinations yielded negative results. The lesions were characterized by involvement of medium and small blood vessels, without any involvement of large vessels. After treatment with steroids and immunosuppressants, the condition improved; however, the possibility of primary CNSV could not be ruled out in some cases [26]. However, none of the seven patients underwent a brain tissue biopsy, and laboratory tests for immunological indicators and viral antibodies produced negative results. Therefore, additional follow-up is still necessary. Since the laboratory characteristics of primary CNSV are unclear, patients may only exhibit vascular lesions, and HR-VWI plays a crucial role in non-invasive testing and evaluation of treatment efficacy [5, 27, 28].

A prevalent reason for arterial ischaemic stroke in children is focal cerebral arteriopathy (FCA), as outlined in previous literature. Unilateral stenosis of intracranial arteries, primarily affecting the anterior circulation, is evident in this condition. HR-VWI in patients with its inflammatory phenotype (FCA-I) indicates segmental stenosis and enhancement of involved arteries [14], which closely resembles the imaging manifested in large and medium vessel vasculitis cases within our group. Previous literature has detailed the imaging appearances of FCA-I without investigating its underlying cause. Our case group of proximal FCA-I comprises a range of systemic immune disorders and infections presenting with neurological manifestations such as stroke, demyelination and inflammatory-like alterations in the brain parenchyma. We contend that FCA-I represents one of the CNSV phenotypes in children, and additional case studies are required to corroborate this notion [29].

Some CNSV are combined with demyelinating lesions as overlapping syndromes, such as SLE combined with neuromyelitis optica spectrum disorders (NMOSDs) or myelin oligodendrocyte glycoprotein antibody-associated disease (MOGAD), and SS combined with NMOSD [30, 31]. Our previous study found that patients with MOGAD may be combined with vasculitis secondary to viral infection [32]. In our cases, three children with CNSV were also positive for demyelinating antibodies (one positive for AQP-4 antibodies and two positive for MOG antibodies), suggesting that the coexistence of vasculitis and CNS demyelinating lesions is more likely to occur in paediatric patients, and there have been previous reports of small vessel vasculitis of the CNS in combination with MOGAD in children, but overlap with NMOSD has not yet been reported and the exact mechanism remains to be investigated [15]. Therefore, we believe that when children with NMOSD and MOGAD present with lesions that have a close vascular relationship, HR-VWI should be supplemented to observe whether there is a combination of CNSV vasculitis [13].

The diagnosis of CNSV in children necessitates a combination of clinical, imaging and laboratory tests. The imaging manifestations of HR-VWI are of immense value. Notably, the imaging differential diagnosis of CNSV in children includes reversible cerebral vasoconstriction syndrome (RCVS), cerebral artery entrapment and atherosclerosis [33, 34]. The primary symptom of RCVS is a thunderclap-like headache. Typically it occurs in the large and medium vessels of the posterior circulation. HR-VWI reveals mild, diffuse thickening of the vessel wall without any enhancement, and in patients with RCVS, vasogenic oedema and ischaemic lesions in the brain parenchyma are often reversible. Additionally, intramural haematoma and torn intima-media sheets of cerebral arterial entrapment may be detected using HR-VWI. However, identifying entrapment in some patients with CNSV is difficult in this case. Atherosclerosis HR-VWI presents with focal plaques and eccentric narrowing of the lumen of the cerebral arteries. This leads to haemorrhage and enhanced activity of the plaque, often accompanied by infarction or haemorrhage of the cerebral parenchyma in the blood-supplying area. It is more commonly observed in middle-aged and elderly individuals, typically with risk factors such as smoking, hypertension and hyperlipidaemia, and is rare in children.

There are limitations in this study. First, the analysis of vasculitis characteristics in children with CNSV of varying aetiologies is preliminary due to the small patient population. A larger sample size may be necessary to eliminate bias. Second, in retrospective studies, the time of HR-VWI examination post-admission and the follow-up interval for patients with CNSV are inconsistent and need improvement in subsequent studies.

## Conclusion

This study underscores the diagnostic value of HR-VWI in CNSV assessment and treatment monitoring, offering a quantitative evaluation of CNSV in children. Assessment of vascular status by HR-VWI provides a good evaluation of CNSV activity and HR-VWI was found to have diagnostic advantages over MRA for CNSV in children.

## Supplementary Material

rkae038_Supplementary_Data

## Data Availability

Data are available upon request.
