# Supplementary material for: Assessment of central nervous system vasculitis in children based on high-resolution vascular wall imaging
Source: Rheumatol Adv Pract. 2024 Mar 7;8(2):rkae038. doi: 10.1093/rap/rkae038 (PMC11009033; doi:10.1093/rap/rkae038)
Supplement: rkae038_Supplementary_Data [file rkae038_supplementary_data.docx]

**
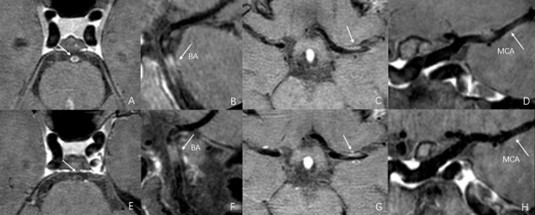
**

**Supplementary Figure S1.** **Female 14-year-old, Neuromyelitis Optica Spectrum Disorders （NMOSD).** Double vision with headache for 10 days was admitted to the hospital with CSF: APQ-4 (+), OCB: IgG (+), lymphocyte and protein were significantly increased. Admission HR-VWI (A-D) showed: BA, left MCA M1 vasculitis, wall thickening with highly intensified luminal stenosis of grade "3" and "1" respectively. The patient was re-examined at 12-month intervals, during which he was regularly taking steroid hormones and immunosuppressants, and his clinical symptoms were relieved. HR-VWI (E-H) showed that the thickening and enhancement of the BA and left MCA M1 vessels had decreased, and the reduction of luminal stenosis was grades "2" and "1", respectively. Re-examination CSF without any abnormalities.

BAA

BAA

**
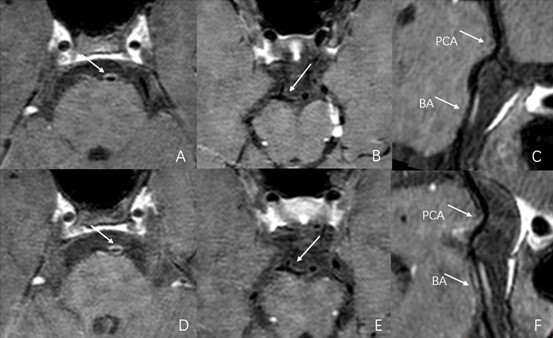
**

**Supplementary Figure S2.** **Female 16-year-old UE.** Left limb numbness and weakness for 10 days was admitted with CSF OCB: IgG (+), low protein levels. HR-VWI before treatment (Figure A, B, C); BA, right PCA P1 segment wall thickening, highly enhanced, lumen stenosis degree of "1" "1" stenosis, respectively. CSF was normal at discharge. There was no regular medication, weakness of the left side of the limb reappeared after an interval of 3 months, and a repeat examination of HR-VWI (Figure D, E, and F) showed progression of vascular lesions, and the degree of luminal stenosis was "2" "1" stenosis, respectively. Re-examination CSF: OCB: IgG (+); Lymphocyte and protein were significantly increased.
